# Editorial: Pharmacology of gangliosides

**DOI:** 10.3389/fphar.2024.1449928

**Published:** 2024-07-18

**Authors:** Hongda Zhuang, Zhendong Huang, Stéphane Birklé, Roger Chammas, Ritva Tikkanen, Yong Chen

**Affiliations:** ^1^ Institute for Advanced Study, Nanchang University, Nanchang, China; ^2^ Nantes Université, University Angers, INSERM, CNRS, CRCI2NA, Nantes, France; ^3^ Center for Translational Research in Oncology, University of São Paulo, São Paulo, Brazil; ^4^ Institute of Biochemistry, Medical Faculty, University of Giessen, Giessen, Germany

**Keywords:** ganglioside, cancer, targeted therapy, diagnostic therapy, immunotherapy, drug delivery

## Introduction

Gangliosides, a large family of glycosphingolipids bearing one or more sialic acid residues, are present on cell surfaces of nearly all vertebrate cells, playing important roles in tissue and organ development and function. Moreover, they exhibit a broad spectrum of functions in various diseases, including cancers (Zheng et al., 2019), neurological disorders (Vasques et al., 2023), infections (Wang et al., 2016), metabolic disorders (Bozic et al., 2024), autoimmune diseases (Inokuchi et al., 2022), and cardiovascular diseases (Ao et al., 2019). Their multifaceted nature positions them as potential targets for therapeutic interventions and diagnostic biomarkers.

To provide an overview on the recent aspects in this field, a Research Topic titled “Pharmacology of gangliosides” was curated. The initiative was led by Professors Dr. Yong Chen from Nanchang University in China, Dr. Roger Chammas from University of Sao Paulo in Brazil, Dr. Ritva Tikkanen from University of Giessen in Germany, and Dr. Stéphane Birklé from Nantes University in France and was published in the journal Frontiers in Pharmacology. This Research Topic includes three review articles and one original research article that are summarized below.

**FIGURE 1:**
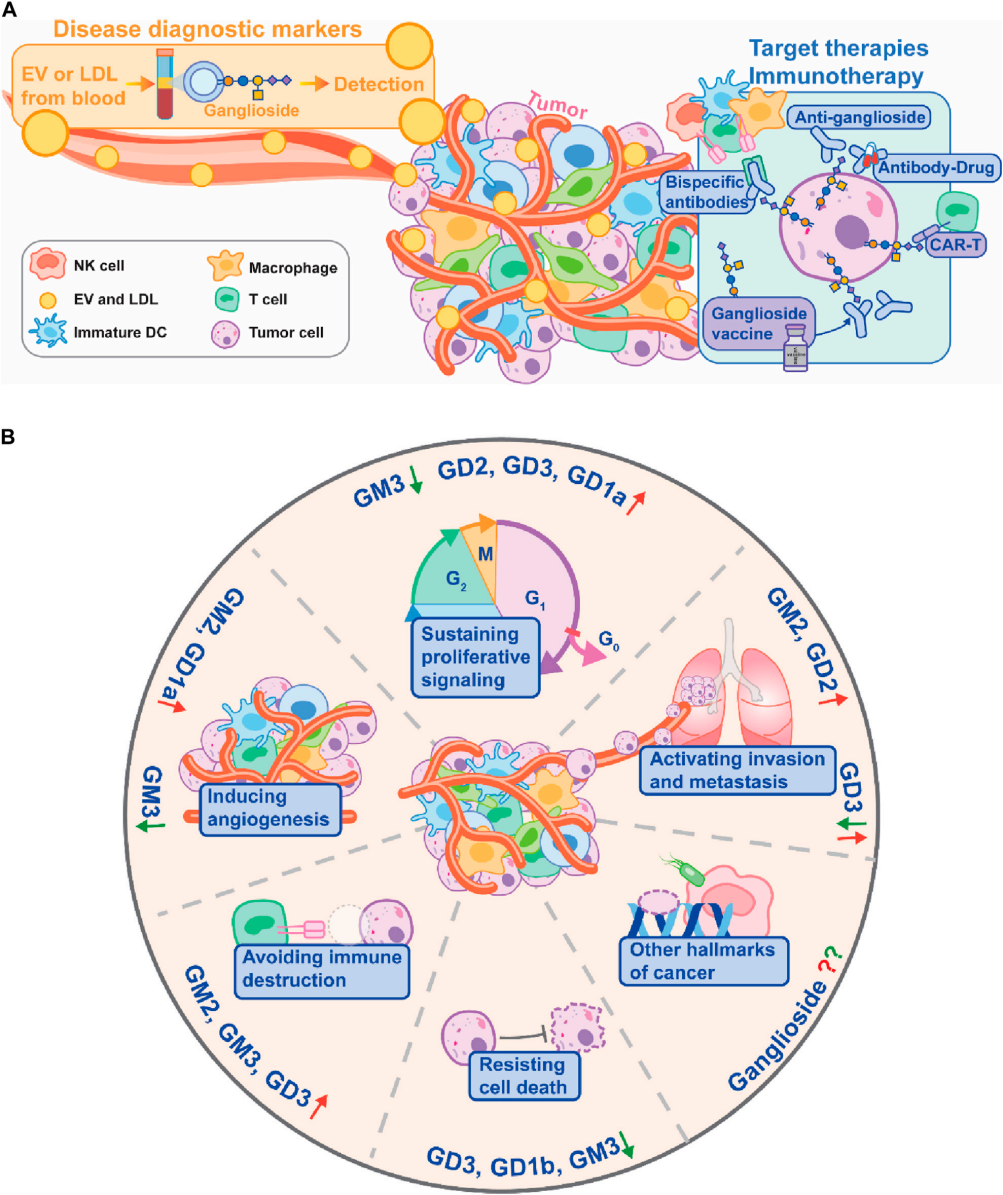
**(A)** Ganglioside Applications in Cancer. This illustration delineates the multifaceted roles of gangliosides in tumor diagnosis, targeted therapy, and immunotherapy. Gangliosides, present on extracellular vesicles (EVs) and LDL, emerge as pivotal tumor diagnostic markers (left upper box). Conversely, gangliosides play instrumental roles in targeted tumor therapy and immunotherapy (right box), serving as targets for bispecific antibodies, antibody-drug conjugates, therapeutic antibodies, and CAR-T cells. Additionally, ganglioside vaccines hold promise in the development of active immunotherapies. **(B)** Influence of Gangliosides on Cancer Hallmarks. Illustration of the positive and negative effects of various gangliosides on different hallmarks of cancer, denoted by upward and downward arrows, respectively. Hallmarks lacking reported information are depicted by question marks, suggesting areas for potential future research. Figure reproduced and modified from Sarkar et al., with permission under the Creative Commons Attribution License (CC BY). ^©^ 2023 Sarkar, Banerjee, and B.iswas.

In the landscape of cancer treatment, formidable challenges endure. Recently, however, ganglioside-related cancer research has upgraded these molecules from mere biomarkers for early tumor diagnosis to novel targets for pharmacological and immunotherapeutic interventions. Our evolving understanding of tumor development mechanisms and treatment strategies has provided breakthroughs in the field of cancer glycobiology. Herein, we discuss pivotal areas of interest cited in this issue (Figure 1A).

## Early diagnostic and prognostic markers

As mentioned above, gangliosides stand out as pivotal cancer biomarkers due to their significantly elevated and consistent levels in both primary and metastatic tumors. Apart from their surface abundance on tumor cells, gangliosides are also shed from the tumor cells into the circulation. As a consequence, tumor-associated gangliosides possess many attributes of circulating tumor biomarkers, providing avenues for early cancer detection and prognostic evaluation (Hayashi et al., 2013; Galan et al., 2023).

In this issue, Nejatie et al. delve into the utilization of tumor associated gangliosides in early detection of cancers. They highlight the biological significance gangliosides and present findings from prior studies on ganglioside abundance, quantification, and detection across various cancers, including melanoma, neuroblastoma, glioblastoma and ovarian cancer. Specifically, they discuss the diagnostic value of GD2 and GD3 in detecting cancers at the early stage, with high specificity and sensitivity. They also propose leveraging a quantitative matrix of the Cancer Biomarker Glycocode and artificial intelligence-driven algorithms to expand the repertoire of validated cancer biomarkers, aiming to address the challenges in cancer diagnosis and to facilitate early intervention (Nejatie et al.).

## Targeted therapies

Gangliosides, as tumor-associated specific antigens, are frequently overrepresented across various cancer types, making them optimal targets for precision therapies. Monoclonal antibody-based therapies directed against gangliosides have demonstrated a notable efficacy in selected patients with neuroblastoma, underscoring their potential in immunotherapeutic interventions (Mabe et al., 2022; Del Bufalo et al., 2023).

Here, Sarkar et al. provide an overview of the multifaceted role of gangliosides in cancer development and progression by influencing critical aspects such as proliferation, epithelial-to-mesenchymal transition, migration, invasion, and immune evasion (Figure 1B; Sarkar et al.). Despite their potential as cancer targets, the structural complexity and functional diversity of gangliosides present significant challenges in research. Immunotherapeutic approaches targeting gangliosides have faced hurdles in clinical trials due to issues like poor immunogenicity and cross-reactivity with normal tissues, causing adverse effects and limiting their efficacy. However, recent findings on selective O-acetylated ganglioside accumulation in cancer tissues offer promising avenues for more specific targeting and reduced side effects. The review underscores the importance of further characterizing O-acetylated gangliosides and developing engineered antibodies for efficient cancer immunotherapies. Additionally, other potential avenues for enhancing the efficacy and the safety of ganglioside immunotherapies are provided (Sarkar et al.).

## Enhanced effect of immunotherapy

Chimeric antigen receptor (CAR) T cell technology represents a groundbreaking advancement that enables precise targeting of tumor cells, bypassing the limitations posed by conventional T cell epitopes, enabling precise targeting of tumor cells. While numerous protein-based tumor-associated antigens have been identified, exploring novel non-protein tumor-associated antigens marks a promising frontier in this field.

Utilizing gangliosides as non-protein CAR targets holds promise for minimizing damage to normal tissues. Among these, GD2 stands out as extensively investigated in CAR-T cell technology, showing promising outcomes in clinical trials. GD2-targeting CAR T cells have demonstrated promising preclinical and clinical efficacy in both neuroblastoma and glioblastoma, (Halliwell et al., 2023). To augment the therapeutic potential of GD2 as a CAR target, combination strategies with other drugs or fusion with other antigens are currently explored (Caforio et al., 2021; Moghimi et al., 2021). As an example, the EZH2 inhibitor tazemetostat has recently been shown to significantly upregulate GD2 level in tumor cells, thereby enhancing CAR-T cell targeting efficacy (Reppel et al., 2022). Similarly, in human lung cancer cells, GM2, another pivotal member of the ganglioside family, has been identified as a non-protein CAR target for CAR-T cell therapy in solid tumors (Sasaki et al., 2023). Other interesting ganglioside targets for CAR-T cell technology include N-glycolyl gangliosides. An example is given by N-glycolyl GM3 (Neu5GcGM3) ganglioside. In human healthy tissue, the predominant form of sialic acid is N-acetyl-neuraminic acid (Neu5Ac), with a more limited presence of N-glycolyl-neuraminic acid (Neu5Gc), due to an inactivating mutation in the cytidine monophosphate N-acetyl-neuraminic acid hydroxylase (CMP-Neu5Ac hydroxylase gene) that prevents the concersion of CMP-Neu5Ac to CMP-Neu5Gc (Irie and Suzuki, 1998). However, abnormal presence of Neu5GcGM3 has been reported in various malignancies. This unexpected presence occurs mainly through the metabolic assimilation of Neu5Gc from a mammalian dietary source (Tangvoranuntakul et al., 2003). Yet, this limited accumulation makes Neu5Gc gangliosides attractive targets for CAR-T cell therapy (Heinzelbecker et al., 2024).

Tumor shed gangliosides have been shown to significantly impair the recognition and elimination of tumor cells by immune cells present within the tumor microenvironment (Kaucic et al., 2006; Liu et al., 2022; Sha et al., 2022; Van Der Haar Àvila et al., 2023). Using ganglioside-specific immunocytokines or bispecific antibodies that bridge tumor cells and immune cells can enhance the patient’s immune system capacity to recognize and attack tumor cells. This targeted approach enhances the efficiency of the immune effector cells against tumor cells and minimizes off-target effects, potentially leading to improved treatment outcomes.

In their review on immunotherapy, Machy et al. provide essential biological insights into GD2 ganglioside, which could help optimizing current immunotherapeutic approaches. Their work underscores the role of GD2 in inducing T cell dysfunction, and its role as an immune checkpoint for macrophages (Machy et al.). Given its involvement in cancer, GD2 has gained considerable interest as a target for cancer immunotherapy. The original study conducted by Bugara et al. highlights the implication of PHLDA1, a protein that they found significantly activated in neuroblastoma cell lines in response to anti-GD2 monoclonal antibody therapy. PHLDA1, previously implicated in regulating cellular differentiation and proliferation, was found to impact the cellular proteome changes and differentiation pathways of human neuroblastoma cells. Their work provides new insights into neuroblastoma management and suggests promising avenues for further research and therapeutic intervention (Bugara et al.).

## Drug delivery systems

Owing to their unique chemical composition and abundant presence on tumor cells, gangliosides serve as attractive candidates for drug delivery systems (Te Welscher et al., 2014). By conjugating drugs with gangliosides, targeted delivery to tumor cells can be achieved, enhancing drug efficacy while minimizing off-target toxicity to normal cells. This approach holds promise for advancing targeted drug delivery strategies in cancer therapy.

The tumor microenvironment typically exhibits acidity, primarily attributed to the accumulation of metabolic byproducts such as lactate and bicarbonate ions, as well as inadequate vascular supply to the tumor. Additionally, the metabolic activity of tumor cells themselves contributes to the generation of acidic metabolites. This acidic milieu promotes tumor cell growth, invasion, and metastasis. GM3-rHDL nanocarriers exhibited targeted delivery to atherosclerotic lesions (acidic milieu). The pH-responsive release of GM3 from GM3-rHDL further amplifies the therapeutic efficacy of exogenous GM3 against atherosclerosis (Rong et al., 2021; Wei et al., 2022). Similar strategy can probably be applied to the treatment of cancers. In addition, Ganglioside GM3 can serve as a drug delivery vehicle by binding to CD169 (Yu et al., 2014), which is a cell surface receptor found on immune cells like dendritic cells and macrophages. It plays a key role in recognizing and clearing pathogens by binding to specific glycosyl molecules on their surface. Consequently, drug delivery vehicles containing GM3 can enhance intracellular drug localization and retention, thus providing a basis for developing tumor vaccines or achieving long-term prevention of viral infections (Eshaghi et al., 2022).

In essence, ganglioside research holds profound significance in the field of cancer. A better understanding of their multifaceted roles remains necessary for the development of effective targeted therapies and immunotherapy. The exploration of the cancer glycocode marks a pivotal step towards the identification of novel biomarkers and candidate antigens for cancer management. Future efforts should delve deeper into elucidating the functions of gangliosides in tumor biology, to develop more effective treatment strategies. Furthermore, while the roles of GD2 or GD3 in cancers have received considerable attention, other gangliosides, such as N-glycolyl GM3 or O-acetyl GD2, have garnered relatively little attention and warrant further fundamental investigation and applied research.
